# Antitumor activity of new chemical compounds in triple negative mammary adenocarcinoma models

**DOI:** 10.2144/fsoa-2019-0057

**Published:** 2020-01-23

**Authors:** María V Giolito, Cristian M Camacho, Maitena Martinez-Amezaga, Carla I Traficante, Rocío A Giordano, Patricia G Cornier, Ernesto G Mata, Carina ML Delpiccolo, Dora B Boggián, Antonela Del Giúdice, Leandro E Mainetti, Olga G Scharovsky, Viviana R Rozados, María J Rico

**Affiliations:** 1 Instituto de Genética Experimental, Facultad de Ciencias Médicas, Universidad Nacional de Rosario, Santa Fe 3100, Rosario 2000, Argentina; 2 Instituto de Química Rosario (CONICET-UNR), Facultad de Ciencias Bioquímicas y Farmacéuticas, Universidad Nacional de Rosario, Suipacha 531, Rosario 2000, Argentina; 3 CONICET (Consejo Nacional de Investigaciones Científicas y Técnicas) CABA (C1425FQB), Argentina; 4 CIUNR (Consejo de Investigaciones, Universidad Nacional de Rosario) Rosario (2000), Argentina

**Keywords:** antitumor effect, chemical agents, *in vitro* assays, triple negative mammary adenocarcinoma

## Abstract

**Aim:**

According to the need for the development of new anticancer agents, we have synthetized novel bioactive compounds and aimed to determine their antitumor action.

**Materials & methods:**

We describe *in vitro* studies evaluating the effect of 35 novel chemical compounds on two triple negative murine mammary adenocarcinoma tumors.

**Results & conclusion:**

Three compounds were selected because of their high antitumor activity and their low toxicity to normal cells. Their effect on tumor cells apoptosis, clonogenicity and migratory capacity, were determined. We found that the selected compounds showed inhibition of viability and clonogenic capacity, and promotion of apoptosis. They also decreased the migratory capacity of tumor cells. The results obtained suggest the likelihood of their future use as antitumor and/or antimetastatic agents.

## Lay abstract

In spite of the important progress achieved in cancer therapeutics, the percentage of people dying because of cancer is still high. Hence, we need to develop new, effective and nontoxic anticancer agents. We synthetized novel compounds and tested their antitumor effect and toxicity, in order to choose those that are effective and do not affect normal cells and therefore, are suitable for human cancer therapies. We selected three out of 35 compounds that show high antitumor action and low toxicity. Also, we studied the mechanisms by which that effect was achieved. Our next goal is to develop experiments with animals in order to have preclinical data that, hopefully, will lead to the clinical use of one or more of the selected compounds.

## Background

Cancer is one of the main causes of morbidity and mortality throughout the world. There were an estimated 18.1 million new cases and 9.6 million cancer deaths worldwide in 2018. Among females, breast cancer is the most commonly diagnosed and is the leading cause of death by cancer [1]. The triple negative (TN) subtype of breast cancer (negative for estrogen, progesterone and HER2/neu receptors) is the most aggressive variety [2]. The absence of the receptors avoids the administration of hormonal and targeted therapies that are useful for other types of breast cancer.

The antitumor therapeutics of the 21st century faces many significant obstacles such as the major adverse effects of the drugs used, the acquired resistance against them and, also, the lack of effective drugs for the treatment of several types of cancer. Hence, there is an urgent need to develop novel antitumor agents that, along with effectiveness, can overcome such disadvantages. The new pharmaceutical agents to be developed must also have a selective action on tumor cells, without affecting normal cells [3].

Recent technological advances in DNA sequencing, cell biology and computer simulation for drug design, have taken the search for new drugs to a higher level [4,5]. While the number of new commercial drugs has not increased as expected, a largely unexplored chemical universe was blamed for that [6,7]. The search for new active compounds cannot rely on just one strategy, and the biological screening of in house libraries is a reliable tool for drug discovery [8]. Previously, a series of synthetic methodologies have been developed, leading to new organic structures, many of which have demonstrated selective antiproliferative activity for certain tumor cell lines [9].

Continuing our search for novel and selective antitumor agents, we herein describe the results of the screening of an in-house synthetic library and its biological evaluation as potential antitumor or antimetastatic compounds.

## Methods

### Chemical compounds

The antitumor effect of 35 compounds belonging to five different families (aminoacyl/peptidyl penicillin derivatives, stilbenes, allenes, β-lactams, oxadiazoles and biaryls; Table 1) was evaluated.

**Table 1.** Library of compounds.

Aminoacyl/peptidyl penicillin derivatives and analogs	Stilbenes	Allenes	β-lactams	Oxadiazoles	Biaryls
PGC1	CIT209aB4	MMA4210f1	CIT171B3	CMC264a	CIT228B5
PGC5	CF30B2	MMA4229f1	CIT75B3	CMC267a	CIT125B1
PGC9	CF29B1		MMA2099f1	CMC266	CIT126B1
PGC11	CF28B2			CMC272c	CIT200B1
PGC17	CF33B2			CMC274c	CIT167B2
PGC18	CF31B1				CIT168B1
PGC21	CIT265B1				CIT219
PGC22i	CIT16e1				
CMC291a	CIT16g1				

Using different methodologies, the compounds were synthesized and purified by column chromatography in the Medicinal Chemistry Laboratory at Instituto de Química de Rosario (IQUIR, NM, Argentina). The methodology and characterization of the chemical compounds are shown in the Supplementary Material 1. The synthesized molecules were characterized by nuclear magnetic resonance and high-resolution mass spectrometry and its purity was checked by thin layer chromatography and Proton Nuclear Magnetic Resonance (^1^H NMR). The compounds were dissolved in DMSO (Anedra, Argentina) and stored at -20°C. All the compounds were diluted in fresh media (Roswell Park Memorial Institute [RPMI, NY, USA] or Dulbecco's Modified Eagle Medium according to the type of cells) before each experiment. Supplementary Material 2 depicts the chemical structures of the compounds belonging to each family.

### Tumors & cell lines

*4T1:* TN murine mammary adenocarcinoma cell line, highly tumorigenic and invasive that can spontaneously metastasize to multiple distant sites.

*MDA-MB-231:* TN human mammary adenocarcinoma cell line.

*MDCK:* Cell line derived from a kidney of an apparently normal adult female cocker spaniel.

4T1 cells were cultured in RPMI supplemented with 10% fetal bovine serum, penicillin (10 U/ml) and streptomycin (10 μg/ml). MDA-MB-231 and MDCK cells were maintained in Dulbecco's Modified Eagle Medium supplemented with 10% fetal bovine serum, penicillin (10 U/ml), streptomycin (10 μg/ml) and L-glutamine (200 nM). All cell types were incubated at 37°C in a 5% CO_2_ atmosphere.

### Chemical compounds screening

**Figure 1.**
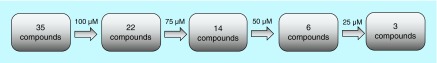
Screening process.

The selection of the compounds with antitumor activity was performed by determining the viability of 4T1 cells incubated with decreasing concentrations of the agents (100, 75, 50 and 25 μM). Those compounds that inhibited cell viability in more than 50% were selected at each concentration and then tested at the following lower concentration (Figure 1).

### Cell viability

The effect of the new chemical compounds on *in vitro* viability was assessed on 4T1 and MDA-MB231 cells (2.5 × 10^3^ cells/well) plated into 96-well culture plates and incubated at 37°C, in a 5% CO_2_ atmosphere. After attachment, different concentrations of the compounds were added, and cells were allowed to grow for 36 h. In order to know their effect on normal cells, MDCK cells (2.5 × 10^5^ cells/well) were plated and treated in the same way. The number of living cells was determined by the tetrazolium salts reduction method (WST-1, Sigma Aldrich, Darmstadt, Germany) as described by the manufacturer. The amount of formazan dye formed, directly correlates with the number of metabolically active cells in the culture. The cell viability was measured in a spectrophotometer (Rayto RT-2100C Microplate Reader, Promega, NC, USA) at 450 nm and expressed as the percentage of control untreated samples. All experiments were performed in triplicate.

### Cell apoptosis analysis

#### Annexin V assay

4T1 cells were seeded into 6-well tissue culture plates until subconfluency. After 18 h of treatment with the selected compounds CIT171B3, CMC266 and PGC22i (25 μM), cells were collected, washed with Annexin buffer (Inmunostep, Spain) and stained with Annexin V-FITC (AP-Biotech, Buenos Aires, Argentina) and propidium iodide (Inmunostep, Salamanca, Spain). Apoptotic rates were determined by flow cytometry (Becton Dickinson FACS Aria II). The percentages of apoptotic cells were calculated using the Flowing software and are the result of three independent experiments.

#### TUNEL assay

4T1 cells were seeded into 6-well tissue culture plates until subconfluency. After 18 h of treatment with the selected compounds CIT171B3, CMC266 and PGC221 (25 μM), they were centrifuged and the pellet obtained was fixed in 10% formalin buffer for 48 h, paraffin-embedded and 5-μm section obtained. Apoptotic cells were identified in the sections using the terminal deoxynucleotidyl transferase-mediated deoxyuridine triphosphate-peroxidase nick end labeling (TUNEL) method (Apoptag^®^ Peroxidase *in situ* Apoptosis Detection Kit, for immunoperoxidase staining, Intergen Company, NY, USA) following the manufacturer instructions. The percentage of TUNEL positive cells with respect to the total number of cells (1000X) of the whole area section, for treated and control cells, were determined.

### Clonogenic assay

4T1 cells (500/well) were plated into 6-well plates. After attachment, they were cultured during 6 days in presence of 10 μM of CIT171B3, CMC266 and PGC22i. After fixing the cells with formalin (4% phosphate buffered saline-buffered paraformaldehyde, Anedra, CABA, Argentina), colonies were stained with Giemsa to allow quantification. Photos of the clones were taken at different times and their size estimated by measuring colonies diameters with the Image J Software. All experiments were performed in triplicate.

### Migration assay

4T1 cells were seeded into 6-well tissue culture plates until subconfluency; then, an artificial wound was generated with a yellow tip and fresh medium was added containing 10 μM of CIT171B3, CMC 266 or PGC 22i (Time 0). Cellular motility was estimated by measuring closure of the initial wound. Photos were taken at 0, 3, 5, 7, 11, 14 and 24 h. Quantification of healing was performed using the Image J software. The percentage of wound healed was calculated using the formula: ([final area/initial area]) × 100 [10].

### Statistical analysis

For the analysis of the data Student's t-test, ANOVA and Tukey-Kramer Multiple Comparison tests, Kruskal–Wallis and Dunn's post-test were used, according to the variable, employing GraphPad Prism version 3.0 (GraphPad Software, USA). p-values lower than 0.05 were considered significant.

## Results

### Compounds screening

#### Antitumor activity in a murine tumor cell line

**Figure 2.**
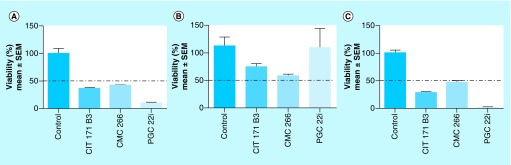
Effect of the compounds in cells viability. Effect on 4T1 **(A)**, MDA-MB-231 **(B)** and MDCK **(C)** cells viability. 4T1, MDA-MB-231 and MDCK cells were incubated for 36 h in complete medium with 25 μM of the compounds. Viable cell number was evaluated with WST-1. Results are shown as percentage of cell viability relative to control (100%) and are expressed as mean ± SEM. Experiments were performed in triplicate. SEM: Standard error of the mean.

**Figure 3.**
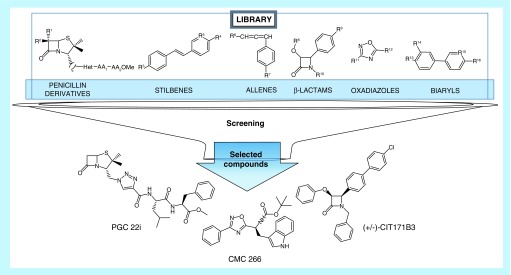
Chemical structure of the selected compounds.

The initial screening of the 35 compounds library, evaluated on 4T1 cells, allowed to select those that showed antitumor action (Figure 1). During the process, there were 22/35 selected chemical compounds at 100 μM (Supplementary Material 3), 14/22 at 75 μM (Supplementary Material 4), 6/14 at 50 μM (Supplementary Material 5A) and 3/6 at 25 μM, the lowest concentration assayed (Supplementary Material 5B). The three selected compounds were: CIT171B3, CMC 266 and PGC 22i. They decreased tumor cells viability in 63.67% ± 1.75 (mean ± standard error of the mean [SEM]; p < 0.05), 58.32% ± 0.94 (p < 0.05) and 89.49% ± 1.08 (p < 0.05), respectively when compared with nontreated control cells (Figure 2A). The percent of viability of 4T1 cells treated with the selected compounds at 25 μM is shown in Supplementary Material 6. The chemical structure of the three compounds is shown in Figure 3.

#### Chemotoxicity

The toxicity of the selected compounds was studied, determining their effect on the viability of MDCK normal cells at 25 μM. CIT171B3 decreased cell viability in 25.65% ± 4.34 (mean ± SEM), CMC266 in 43.06% ± 2.34 and, interestingly, PGC22i in 14.69% ± 14.69, with respect to nontreated control cells (Figure 2B).

#### Antitumor activity in a human cancer cell line

Importantly, the selected compounds showed antitumor activity when tested at 25 μM on the human breast cancer cell line MDA-MB-231. CIT171B3, CMC266 and PGC22i decreased tumor cells viability in 71.46% ± 1.26 (mean ± SEM; p < 0.01), 54.02% ± 2.56 (p < 0.01) and 98.43% ± 0.31 (p < 0.01), respectively, when compared with nontreated control cells (Figure 2C).

### Apoptosis

**Figure 4.**
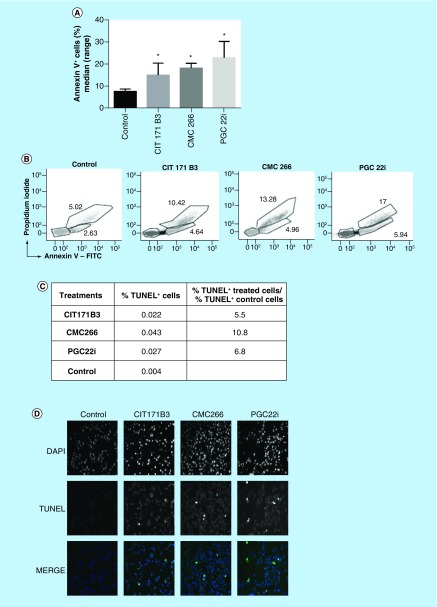
Apoptosis induced by the treatment. **(A)** After 18 h of treatment with the compounds (25 μM), 4T1 cells were collected, washed and stained with Annexin V-FITC and propidium iodide. It shows the percentage of Annexin V^+^ cells (median [range] of three independent experiments); (Mann–Whitney test) compared with control cells; **(B)** Flow cytometry profiles for each compound; **(C)** After 18 h of treatment with the selected compounds CIT171B3, CMC266 and PGC22i (25 μM), apoptotic cells were identified in sections of paraffin-embedded cells, using terminal deoxynucleotidyl transferase-mediated deoxyuridine triphosphate-peroxidase nick end labeling assay. The table shows the ratio between the percentage of terminal deoxynucleotidyl transferase-mediated deoxyuridine triphosphate-peroxidase nick end labeling positive-treated cells and the percentage obtained in nontreated control cells; **(D)** Photos representative of each treatment 1000X. *p < 0.05. DAPI: 4′,6-diamidino-2-phenylindole; TUNEL: Terminal deoxynucleotidyl transferase-mediated deoxyuridine triphosphate-peroxidase nick end labeling.

**Figure 5.**
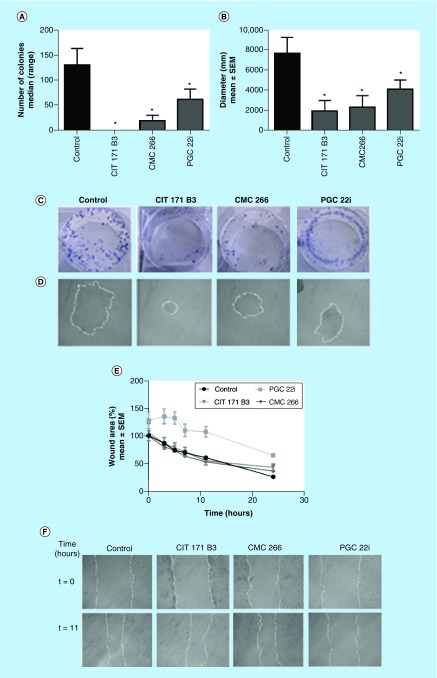
Effect on clonogenic and migratory capacity. Treatment with 10 μM (day 6): **(A)** Number of colonies (median [range]; Mann–Whitney test); **(B)** Diameter in mm (mean ± standard error of the mean of three independent experiments; Student's t-test). Representative images of: **(C)** Colonies (Giemsa staining); **(D)** Colony morphology taken under inverted microscope (100×); **(E)** Cell motility was estimated by measuring closure of the initial wound (% respect to the initial wound) incubating with the compounds (10 μM). Quantification was performed using the Image J software and data are shown as median (range) of three independent experiments; **(F)** Photos taken at 0 and 11 hs (100×). *p < 0.05. SEM: Standard error of the mean.

The percentage of Annexin V^+^ cells, when incubated with 25 μM of CIT171B3 (11.9% [7.64–25.62]; median [range]), CMC266 (17.9% [14.68–22.18]) and PGC22i (25.8% [8.94–34.1]) was significantly higher (p < 0.05) than that of nontreated control cells (6.6% [5.46–11.91]; Figure 4A). The individual flow cytometry profiles are shown in Figure 4B. Moreover, when apoptosis was determined with the TUNEL assay, CIT171B3, CMC266 and PGC22i increased 5.5, 10.8 and 6.8-times, respectively, the number of TUNEL positive cells, with respect to nontreated control cells (Figure 5C & D).

### Clonogenic assay

In order to avoid possible bias in the results, because of the already demonstrated effect of the compounds on apoptosis at a concentration of 25 μM, the clonogenic assay was developed at a lower concentration. When tested at 10 μM the number of colonies of 4T1 cells, when compared with the control (median [range], 130 [85–163]) was lower for CIT171B3 0 [0–0] p = 0.0286, CMC266 (19 [0–30]) p = 0.0286 and PGC22i (61 [11–82]) p = 0.0286 (Figure 5A). Also, the compounds diminished the diameter of the colonies (mm, mean ± SEM) CIT171B3: 1931 ± 1034, p < 0.05; CMC266: 2293 ± 11.57, p = 0.0505 and PGC22i: 4087 ± 923.5, p = 0.0597 with respect to control (7678 ± 1565; Figure 5B). In Figure 5C & D are shown images of stained colonies and their morphology.

### Migratory capacity

The assay to determine the effect of each selected compound on the migratory capacity of the cells was also developed at 10 μM. 4T1 cells showed a lower migratory capacity with respect to control cells when cultured with 10 μM of CIT171B3 and CMC266 (p < 0.01 at 24 h), and PGC22i (p < 0.01 at 3, 5, 7, 11 and 24 h; Figure 5E). Representative photos, taken at 0 and 11 hs with a 100× magnification, are shown in Figure 5F.

## Discussion

In Argentina, the cancer incidence rate is medium–high (212 cases per 100,000 inhabitants), with breast cancer showing the highest incidence in women, followed by male prostate and lung cancers [11]. They represent a global public health concern [12] considering that, worldwide, they are the leading causes of death from cancer.

Among the different types of breast cancer, the TN subtype shows high aggressiveness and risk of recurrences hence, it is associated with poor prognosis. Patients with this type of tumor have scarce therapeutic options, a fact that underlines the need of finding new treatments endowed of high efficacy and tolerability.

Nowadays, the process of drug discovery and its development has shifted gears. Thousands of products differing in their chemical structure are tested for their activity against different molecular targets [13]. In the field of oncology, the main objective is the development of drugs with high antitumor efficacy and low toxicity to normal cells [3].

The problems generated by the drugs for cancer treatment such as toxicity, as well as generation of drug resistance, among others, are fueling the search by both academy and industry, of novel and better chemotherapeutic agents [9,14]. In the last decade, there was a significant decrease in the rate at which new candidate drugs were translated into effective clinical therapies. Specifically, there was an increase in the removal of drugs that were already in Phase II or III, mainly due to the lack of efficacy and clinical safety or to the presence of toxic effects, which caused 30% of failures [3].

The already mentioned issues of adverse effects and drug resistance of established antitumor agents, together with the scarcity of effective therapies for some types of cancer, pose an important problem for public health [15,16]. Due to these difficulties, the development of new improved chemotherapeutic agents, as well as new schemes of administration, is a constant challenge for the industry and the academic world [9]. Because of this need, the Medicinal Chemistry Laboratory at IQUIR has been developing methodologies for the synthesis of potential bioactive compounds such as penicillin/amino acid hybrids. Based on structure–activity studies of penicillin/amino acid hybrids, a series of analogs were identified showing inhibition of cell proliferation against certain tumor cell lines, without generating toxicity to normal cells [9,17,18]. Also, other synthetic compounds, from different chemical families, were generated. Hence, it was interesting to evaluate the *in vitro* antitumor effect of this in-house library of new synthesized chemical compounds, which belonged to the following families: aminoacyl/peptidyl penicillins, oxadiazoles, β-lactams, stilbenes, biaryls and allenes.

At 100 μM the best antitumor effect was obtained with triazolyl peptidyl penicillins, while compounds bearing only one amino acid (PGC1), having an oxadiazol ring instead of a triazole (CMC2911a), or lacking the penicillin moiety (PGC9), did not show inhibition of cell viability. Among the aryl-oxadiazolyl amino acids, only those bearing Leu (CMC267a) and Trp (CMC266) yielded tumor cells viability below 50%. In contrast, all the monocyclic β-lactams were active at that concentration. Interestingly, most of the stilbenes (4-styrylbenzoic acid derivatives) gave a good decrease in cells viability. Clearly, only the stilbene bearing a free carboxylic acid (CIT265B1) showed low antitumor activity. Similarly, biaryl derivatives were mostly active, the most active being those bearing a carboxylic ester group (CIT228B5, CIT125B1 and CIT126B1). Finally, allenes (MMA4210f1 and MMA4229f1) were not active, even at 100 μM.

At 75 μM, all selected triazolyl peptydyl penicillins showed a decrease in cell viability greater than 50%, while the monocyclic β-lactam bearing a boronic acid functionality (MMA2099f1) was discarded due to its low effect on the tumor cell. Stilbenes were inactive and most of the biaryl derivatives were selected, although the decrease in cell viability was just behind 50% in most of the cases. As expected, at 50 μM biaryl and stilbenes showed cell viability higher than 50%, leaving three triazolyl peptydyl penicillins and two aryl-oxadiazolyl amino acid and a 4-chloro, 4-biaryl β-lactam for further assays. Interestingly, leucine/valine and tryptophan are a common feature among the selected amino acid-containing penicillins and oxadiazolyl derivatives. Finally, three compounds were selected at 25 μM: CIT171B3 and CMC266 showed similar inhibition of viability (near 60%), while PGC22i evidenced a stronger effect on the same type of cells (89%). These compounds belonged to different families: β-lactams: CIT171B3, oxadiazoles: CMC266 and aminoacyl/peptidyl penicillins: PGC22i. Thus, the aforementioned selection process of the 35 compounds library allowed to identify three compounds that exerted antitumor action at the lowest concentration assayed.

The presence of mild or null toxicity on normal cells caused by the compounds is an essential condition for future translation of the results to the *in vivo* setting. Consequently, the toxicity of the three selected compounds was assayed using the MDCK normal cell line. All of them affected viability with different intensities always being higher than 50%. At 25 μM, CIT171B3 and CMC266 decreased cell viability in 20 and 40%, respectively, while PGC22i did not modify it at all (Figure 2B).

Another important issue related to a putative translation to the clinics is the activity of the compounds on cells of human origin. Hence, the next step was to verify their action on the TN human MDA-MB-231 breast cancer cell line. The significant decrease in the viability of the human cancer cells, caused by the lowest concentration tested of the selected compounds, reaching values as high as 98% for PGC22i, are encouraging. The results obtained in cells belonging to different species, rodent and human, strengthens the probability of their utility as anticancer agents.

In order to know the mechanisms by which the compounds could exert their antitumor effect, we analyzed their ability to modify different properties of the tumor cells. The evaluation of the apoptosis by Annexin V assay induced by the selected compounds, demonstrated that all of them significantly increased the percentage of apoptotic 4T1 cells (Figure 4A & B), indicating their action favored programmed cell death. Also, in agreement with the mentioned results, the TUNEL assay showed a high increase in apoptosis in the cells treated with the compounds with respect to that observed in control cells (Figure 4C & D).

Moreover, by means of the clonogenic assay, it was evaluated the effect of the treatment on cell reproductive death and, consequently, the fraction of seeded cells that can proliferate indefinitely hence, develop colonies. It was demonstrated that at 10 μM, the three compounds significantly inhibited the number of colonies (Figure 5A), and diminished their diameters, being that decrease significant for CIT171B3 and PGC22i (Figure 5B).

During the metastatic process, malignant cells undergo the epithelial-mesenchymal transition, a process that enables them to invade and colonize neighboring and/or distant tissues. In order to evaluate one of the events that are part of the metastatic cascade, we assessed the ability of the compounds to inhibit cell migration by the wound healing assay. CIT171B3, CMC266 and PGC22i decreased the migratory capacity of 4T1 cells at 10 μM when compared with nontreated control cells, an effect that began earlier and was stronger for PGC22i (Figure 5E).

The β-lactam (azetin-2-one) ring is considered a privileged structure since it is present in compounds with diverse biological activity. Apart from the classical antibiotic effect of penicillins, cephalosporins and monobactams [19], this ring is found, for example, in potent cholesterol absorption inhibitors, including the commercial drug ezetimibe [20]. In the field of antiproliferative chemotherapy, several β-lactams derivatives have shown activity against cancer cells. Two of the selected compounds are β-lactams: PGC22i is a penicillin and CIT171B3 is a monobactam. Both, penicillin compounds [9,17,21] and monobactams [22–24], have demonstrated antitumor activity.

As reported in the literature, oxadiazoles have demonstrated an evident antitumor activity. Oxadiazoles are heterocyclic compounds with a broad spectrum of biological activities among which, antiproliferative and proapoptotic capacities have been demonstrated for human breast cancer cell lines [25]. In agreement with those results, the new compound CMC266, which belong to the oxadiazoles family herein studied evidenced cytotoxic action against the mammary tumor cells.

## Conclusion

The antitumor activity of an in house synthetic library was evaluated. From them, three compounds were selected for further studies. They were an aminoacyl/peptidyl penicillin (PGC22i), an aryl-oxadiazolyl amino acid (CMC266) and a 4-chloro,4-biaryl β-lactam (CIT171B3). The selected compounds showed, with different intensities on different cell lines, inhibition of viability, promotion of apoptosis and inhibition of clonogenic capacity. Results showed that the aminoacyl/peptidyl penicillin (PGC22i) exhibited the most effective antitumor action together with the interesting and necessary characteristic of lack of toxicity for normal cells. The inhibition of tumor growth by PGC22i could be due, at least in part, to both decrease cell proliferation and increase of cell death by apoptosis. Also, a promising effect of this compound on cells motility is related to their capacity to disseminate and metastasise, suggesting a putative antimetastatic action. The aforementioned properties warrant its *in vivo* testing in preclinical studies, which may lead to its clinical testing.

## Future perspective

The number of drug-like structures theoretically available by synthesis is more than 970 million, this fact making organic synthesis an almost unlimited source of new and diverse small molecules for drug discovery.

The possibility of designing and testing drug-like compounds with a probable biological activity is supported by the analysis of structure–activity relationships. Therefore, it is expected that the number of compounds with antitumor and/or antimetastatic activity will increase in the near future, taking the search of new therapeutic approaches to a higher level.

**Summary points**

- Organic compounds evaluated here were synthetized by solution-phase as well as solid-phase methodologies. The latter is a technique that complements the former, allowing the generation of interesting structures in a rapid and efficient manner. Solid-phase synthesis also contributes to green chemistry by reducing chromatographic separations and, consequently, decreasing organic solvent waste.
- This work deals with the treatment of mammary/breast adenocarcinomas, the most common cancer in women all over the world and, particularly, with the most aggressive triple negative subtype. Triple negatve breast cancer lacks the expression of estrogen, progesterone and HER2/neu receptors, hence reducing the opportunities of hormonal or targeted therapies.
- The cell lines used for determining the antitumor effect of the compounds were not only of murine but also of human origin, moving, therefore, a step closer to their clinical application.
- Thirty-five compounds were studied and, among them, three were selected. They were chosen because of their high antitumor activity and low toxicity for normal cells.
- The selected compounds were an aminoacyl/peptidyl penicillin (PGC22i), an aryl-oxadiazolyl amino acid (CMC266) and a 4-chloro,4-biaryl β-lactam (CIT171B3). The selected compounds showed, with different intensities on different cell lines, inhibition of viability, promotion of apoptosis and inhibition of clonogenic capacity. Results showed that the aminoacyl/peptidyl penicillin (PGC22i) exhibited the most effective antitumor action together with the interesting and necessary characteristic of lack of toxicity for normal cells.
- Interestingly, PGC22i, also demonstrated the highest inhibition on cell motility, a property that tumor cells need to be able to disseminate to other sites and to continue with the metastatic cascade. Therefore, it suggests a putative antimetastatic action.
- The aforementioned properties warrant the *in vivo* testing of PGC22i in preclinical studies, which may lead to its clinical testing.
