# Supplementary material for: Antitumor activity of new chemical compounds in triple negative mammary adenocarcinoma models
Source: Future Sci OA. 2020 Jan 23;6(3):FSOA442. doi: 10.2144/fsoa-2019-0057 (PMC7050605; doi:10.2144/fsoa-2019-0057)
Supplement: Supplementary file 1 [file fsoa-06-442-s1.docx]

| **General Structure: Aminoacyl/peptidyl**  **penicillin derivatives** | **code** | **R^1^** | **R^2^** | **n** | **Het** | **AA_1_** | **AA_2_** |
| --- | --- | --- | --- | --- | --- | --- | --- |
|  | PGC1 | Br | Br | 1 |  | Gly | - |
|  | PGC5 | Br | Br | 1 |  | Tyr | Leu |
|  | PGC9 | Br | Br | 1 |  | CO_2_CH_2_Ph | - |
|  | PGC11 | Br | Br | 1 |  | Phe | Val |
|  | PGC17 | Br | Br | 1 |  | Phe | Met |
|  | PGC18 | Br | Br | 1 |  | Trp | Leu |
|  | PGC22i | H | H | 1 |  | Phe | Leu |
|  | CMC291a | Br | Br | 0 |  | 4-BrPh | - |
|  | PGC21 | - | - | - |  | Phe | Leu |

| **General Structure: Stilbenes** | **code** | **R^3^** | **R^4^** | **R^5^** |
| --- | --- | --- | --- | --- |
|  | CIT209aB4 | COOMe | NEt_2_ | CH |
|  | CF30B2 | COOMe | CHO | CH |
|  | CF29B1 | COOMe | Cl | CH |
|  | CF28B2 | COOMe | H | N |
|  | CF33B2 | COOMe | H | COMe |
|  | CF31B1 | COOMe | NO_2_ | CH |
|  | CIT265B1 | COOH | OH | CH |
|  | CIT16e1 | COOMe | Cl | CH |
|  | CIT16g1 | COOMe | CF_3_ | CH |

| **General Structure: Allenes** | **code** | **R^6^** | **R^7^** |
| --- | --- | --- | --- |
|  | MMA4210f1 | C_8_H_17_ | CO_2_Me |
|  | MMA4229f1 | C_8_H_17_ | Me |

| **General Structure: β-lactams** | **code** | **R^8^** | **R^9^** | **R^10^** |
| --- | --- | --- | --- | --- |
|  | CIT171B3 | Ph | 4-Cl-Ph | Bn |
|  | CIT75B3 | Ph | 4-OMe-Ph | Bn |
|  | MMA2099f1 | Ph | 4-B(OH)_2_-Ph | Bn |

| **General Structure: Oxadiazoles** | **code** | **R^11^** | **R^12^** |
| --- | --- | --- | --- |
|  | CMC264a | Ph |  |
|  | CMC267a | Ph |  |
|  | CMC266 | Ph |  |
|  | CMC272c | 4Br-Ph |  |
|  | CMC274c | 4Br-Ph |  |

| **General Structure: Biaryls** | **code** | **R^13^** | **R^14^** | **R^15^** | **R^16^** |
| --- | --- | --- | --- | --- | --- |
|  | CIT228B5 | COOCH_3_ | H | N | H |
|  | CIT125B1 | COCH_3_ | H | COCH_3_ | H |
|  | CIT126B1 | COCH_3_ | H | CH | Cl |
|  | CIT200B1 | H | H | CH | NEt_2_ |
|  | CIT167B2 | H | OCH_3_ | N | H |
|  | CIT219 | OCH_3_ | H | CH | NEt_2_ |
|  | CIT168B1 | H | OCH_3_ | - | - |
