# Supplementary material for: Antitumor activity of new chemical compounds in triple negative mammary adenocarcinoma models
Source: Future Sci OA. 2020 Jan 23;6(3):FSOA442. doi: 10.2144/fsoa-2019-0057 (PMC7050605; doi:10.2144/fsoa-2019-0057)
Supplement: Supplementary file 2 [file fsoa-06-442-s2.docx]

**Methodology and characterization of chemical compounds**

Chemical reagents were purchased from commercial sources and were used without further purification unless noted otherwise. Solvents were analytical grade or were purified by standard procedures prior to use. Compounds PGC1,^1^ PGC5,^1^ PGC9,^1^ PGC11,^1^ PGC17,^1^ PGC18,^1^ PGC22i,^1^ PGC21,^1^ CIT209aB4,^2^ CF30B2,^2^ CF29B1,^2^ CF28B2,^2^ CF33B2,^2^ CF31B1,^2^ CIT265B1,^2^ MMA4229f1,^3^ CIT171B3,^4^ CIT75B3,^4^ CMC264a,^5^ CIT228B5,^6^ CIT125B1,^4^ CIT126B1,^4^ CIT200B1,^4^ CIT167B2,^4^ CIT219,^4^ CIT16e1,^4^ CIT16g1,^6^ CIT168B1,^6^ have been already described.

**Synthesis of (2*S*)-6,6-dibromo-2-(3-(4-bromophenyl)-1,2,4-oxadiazol-5-yl)-3,3-dimethyl-4-thia-1-azabicyclo[3.2.0]heptan-7-one (CMC291a)**

6,6-dibromopenicillanic acid **1** (1 eq) was activated by HATU (1 eq) / HOBt (0.2 eq) in the presence of DIPEA (5 eq) for 1 h at room temperature, using dichloromethane as solvent. Then, amidoxime **2** (1 eq) was added and the reaction was heated to 120°C for 2 h in a mixture of toluene/dichloromethane (1:1) to obtain the crude compound CMC291a, which was purified by chromatographic column (15% yield). ^1^H NMR (CDCl_3_, 300 MHz) δ (ppm): 1.30 (s, 3H, CH_3_), 1.75 (s, 3H, CH_3_), 5.36 (s,1H, CH), 6.00 (s,1H, CH), 7.64 (d, 2H, ArH), 7.96 (d, 2H, ArH). ^13^C NMR (CDCl_3_, 75 MHz) δ (ppm): 25.59, 33.23, 58.47, 64.66, 65.57, 80.78, 124.87, 126.36, 129.08, 132.30, 164.34, 167.83, 173.48.

**Synthesis of (4-(1-benzyl-4-oxo-3-phenoxyazetidin-2-yl)phenyl)boronic acid (MMA2099f1)**

Benzylamine (**3**) (64 mg, 0.6 mmol) and (4-formylphenyl)boronic acid (**4**) (112.5 mg, 0.75 mmol) were stirred at 0ºC for 1 hour in anhydrous DMF with activated MS 4Å. The intermediate **5** was dissolved in anhydrous DCM at 0ºC, 2-phenoxyacetyl chloride (**6**) (153 mg, 0.9 mmol) and anhydrous TEA (0.3 mL, 2.1 mmol) were added and the reaction mixture was stirred overnight at room temperature. The product **MMA2099f1** was isolated with 56% yield after column chromatography (40/60-hexane/AcOEt). ^1^H NMR (CDCl_3_, 300 MHz) δ (ppm): 3.85 (d, *J* = 14.7 Hz, 1H), 4.77 (d, *J* = 4.4 Hz, 1H), 4.89 (d, *J* = 14.8 Hz, 1H), 5.42 (d, *J* = 4.5 Hz, 1H), 6.85 (m, 1H), 6.99 – 7.18 (m, 5H), 7.27 – 7.43 (m, 6H), 7.71 (d, *J* = 8.1 Hz, 2H). ^13^C NMR (CDCl_3_, 75 MHz) δ (ppm): 44.4, 61.5, 82.1, 115.5, 122.1, 128.2, 128.7, 128.9, 128.9, 129.3, 133.7, 134.5, 135.5, 156.8, 165.9.

**Synthesis of 4-(undeca-1,2-dien-1-yl)phenyl acetate (MMA4210f1)**

Methyl 4-(1-(pyrrolidin-1-yl)undec-2-yn-1-yl)benzoate (**7**) (synthesized in our laboratory, 23 mg, 0.065 mmol) was added to a MW vessel, followed by the addition of AgNO_3_ (6 mg, 0.033 mmol) and MeCN (1,5 mL). The reaction vessel was capped and introduced in the MW reactor for 60 minutes at 120ºC (settings: fast stirring, 200W=max. power, closed-vessel system). After column chromatography (98/2-hexane/AcOEt), the product **MMA4210f1** was isolated as a colourless oil, the yield was 66%. ^1^H NMR (CDCl_3_, 300 MHz) δ (ppm): 0.80 – 0.93 (m, 3H), 1.21 – 1.38 (m, 10H), 1.42 – 1.52 (m, 2H), 2.14 (qd, *J* = 7.0, 3.0 Hz, 2H), 3.90 (s, 3H), 5.62 (q, *J* = 6.7 Hz, 1H), 6.11 – 6.18 (m, 1H), 7.33 (d, *J* = 8.4 Hz, 2H), 7.96 (d, *J* = 8.4 Hz, 2H). ^13^C NMR (CDCl_3_, 75 MHz) δ (ppm): 14.1, 22.7, 28.5, 29.1, 29.2, 29.3, 29.4, 31.8, 52.0, 94.2, 95.5, 126.4, 128.1, 129.9, 140.3, 167.0, 206.3.

**Syntheses of 1,2,4-oxadiazoles CMC267a, CMC266, CMC272c, CMC274c**

Different Boc-protected amino acids (**8**) in DMF were activated using HATU (1eq) / HOBt (0.2 eq) in the presence of DIPEA (5eq), for 1 min. at room temperature; then the amidoxime **9** was added and stirred for 1 h at room temperature to obtain the acylated intermediate **10**. The system was heated at reflux for 2 h in the same solvent, to achieve cyclization, obtaining the 1,2,4-oxadiazole **11**.

***tert*-Butyl (*S*)-(3-methyl-1-(3-phenyl-1,2,4-oxadiazol-5-yl)butyl)carbamate (CMC267a):** ^1^H NMR (CDCl_3_, 300 MHz) δ (ppm): 0.97 (d, 3H, J=2.19 Hz, CH_3_ Leu), 0.99 (d, J= 2.19 Hz, CH_3_ Leu),1.44 (s, 9H, Boc CH_3_), 1.74-1.81 (m, 3H, CH y CH_2_ Leu), 5.11 (m, 1H), 4.94-4.95 (bs,1H, NH), 7.47 (m, 3H, ArH), 8.07 (m, 2H, ArH). ^13^C NMR (CDCl_3_, 75 MHz) δ (ppm): 21.98, 22.55, 24.69, 28.20, 43.23, 46.79, 80.41, 126.63, 127.49, 128.79, 131.20, 154.96, 168.26, 180.01.

***tert*-Butyl (*S*)-(2-(1H-indol-3-yl)-1-(3-phenyl-1,2,4-oxadiazol-5-yl)ethyl)carbamate (CMC266):** ^1^H NMR (CDCl_3_, 300 MHz) δ (ppm): 1.45 (s, 9H, Boc CH_3_), 3.50 (d, *J*= 3.98 Hz, Trp CH_2_), 5.29-5.39 (m, Trp CH), 5.46 (bs, NH), 6.87 (bs, indole ring NCH), 7.07-7.19 (m, 2H, indole ring),7.25-7.32 (m, 1H, indole ring), 7.46-7.54 (m, ArH 3 H, indole 1H), 8.04 (d, *J*= 6.24, 2H, ArH), 8.27 (bs, indole NH). ^13^C NMR (CDCl_3_, 75 MHz) δ (ppm): 28.30, 49.47, 80.51, 109.20, 119.79, 122.29, 123.13, 126.60, 127.52, 128.86, 131.28, 136.14, 155.07, 168.28, 179.35.

***tert*-Butyl (*S*)-(1-(3-(4-bromophenyl)-1,2,4-oxadiazol-5-yl)ethyl)carbamate (CMC272c):** ^1^H NMR (CDCl_3_, 300 MHz) δ (ppm): 1.45 (s, 9H, Boc CH_3_), 1.62 (d, 3H, *J*= 6.84 Hz, Ala CH_3_),5.15 (bs, 1H CH, 1H NH), 7.61 (d, 2H ArH, *J*= 8.59 Hz), 7.94 (d, 2H ArH, *J*= 8.59 Hz). ^13^C NMR (CDCl_3_, 75 MHz) δ (ppm): 20.03, 28.28, 44.28, 80.61, 125.55, 125.85, 128.98, 132.15, 154.74, 167.60, 180.34.

***tert*-Butyl (*S*)-(1-(3-(4-bromophenyl)-1,2,4-oxadiazol-5-yl)-3-methylbutyl)-carbamate (CMC274c):** ^1^H NMR (CDCl_3_, 300 MHz) δ (ppm): 0.97 (d, 3H, J=2.13 Hz, CH_3_ Leu), 1.00 (d, 3H, J=2.16 Hz, CH_3_ Leu), 1.44 (s, 9H, Boc CH_3_), 1.71-1.77 (m, 3H, CH y CH_2_ Leu), 4.77-5.12 (m, 1 H Leu CH, 1H NH), 7.61 (d, 2H ArH, J= 8.55 Hz), 7.93 (d, 2H ArH, J= 8.55 Hz). ^13^C NMR (CDCl_3_, 75 MHz) δ (ppm): 20.91, 21.54, 21.96, 27.25, 42.16, 45.80, 79.51, 124.60, 124.64, 127.98, 131.10, 153.94, 166.57, 179.29.

**References:**

1.-Cornier PG, Delpiccolo CM, Mascali FC, Boggián DB, Mata EG, Cárdenas MG, Blank VC, Roguin LP. In vitro anticancer activity and SAR studies of triazolyl aminoacyl (peptidyl) penicillins. *MedChemComm*, *5*(2), 214-218 (2014).

2.-Traficante CI, Fagundez C, Serra GL, Mata EG, Delpiccolo, CML. Chemoselective and sequential palladium-catalyzed couplings for the generation of stilbene libraries via immobilized substrates. *ACS combinatorial science*, *18*(5), 225-229 (2016).

3.-Lü R, Ye J, Cao T, Chen B, Fan W, Lin W, Liu J, Luo H, Miao B, Ni S, Tang X, Wang N, Wang Y, Xie X, Yu Q, Yuan W, Zhang W, Zhu C, Ma S. Bimetallic enantioselective approach to axially chiral allenes. *Organic letters*, *15*(9), 2254-2257 (2013).

4.-Traficante CI, Mata EG, Delpiccolo CM. Very efficient and broad-in-scope palladium-catalyzed Hiyama cross-coupling. The role of water and copper (i) salts. RSC Advances, *5*(34), 26796-26800 (2015).

5.-Braga VML, Melo SJD, Srivastava RM, Falcão EPDS. Synthesis of new 1, 2, 4-oxadiazoles carrying (1'S, 2'S)-t-butyloxycarbonyl-1-amino-2-methyl-1-butyl and (1'S)-t-butyloxycarbonyl-1'-amino-1'-ethyl groups at C-5. *Journal of the Brazilian Chemical Society*, *15*(4), 603-607 (2004).

6.-Traficante CI, Delpiccolo CML, Mata EG. Palladium-Catalyzed Cross-Coupling Reactions of Arylsiloxanes with Aryl Halides: Application to Solid-Supported Organic Synthesis. *ACS combinatorial science*, *16*(5), 211-214 (2014).
