# Supplementary figures and images for: Antitumor activity of new chemical compounds in triple negative mammary adenocarcinoma models
Source: Future Sci OA. 2020 Jan 23;6(3):FSOA442. doi: 10.2144/fsoa-2019-0057 (PMC7050605; doi:10.2144/fsoa-2019-0057)

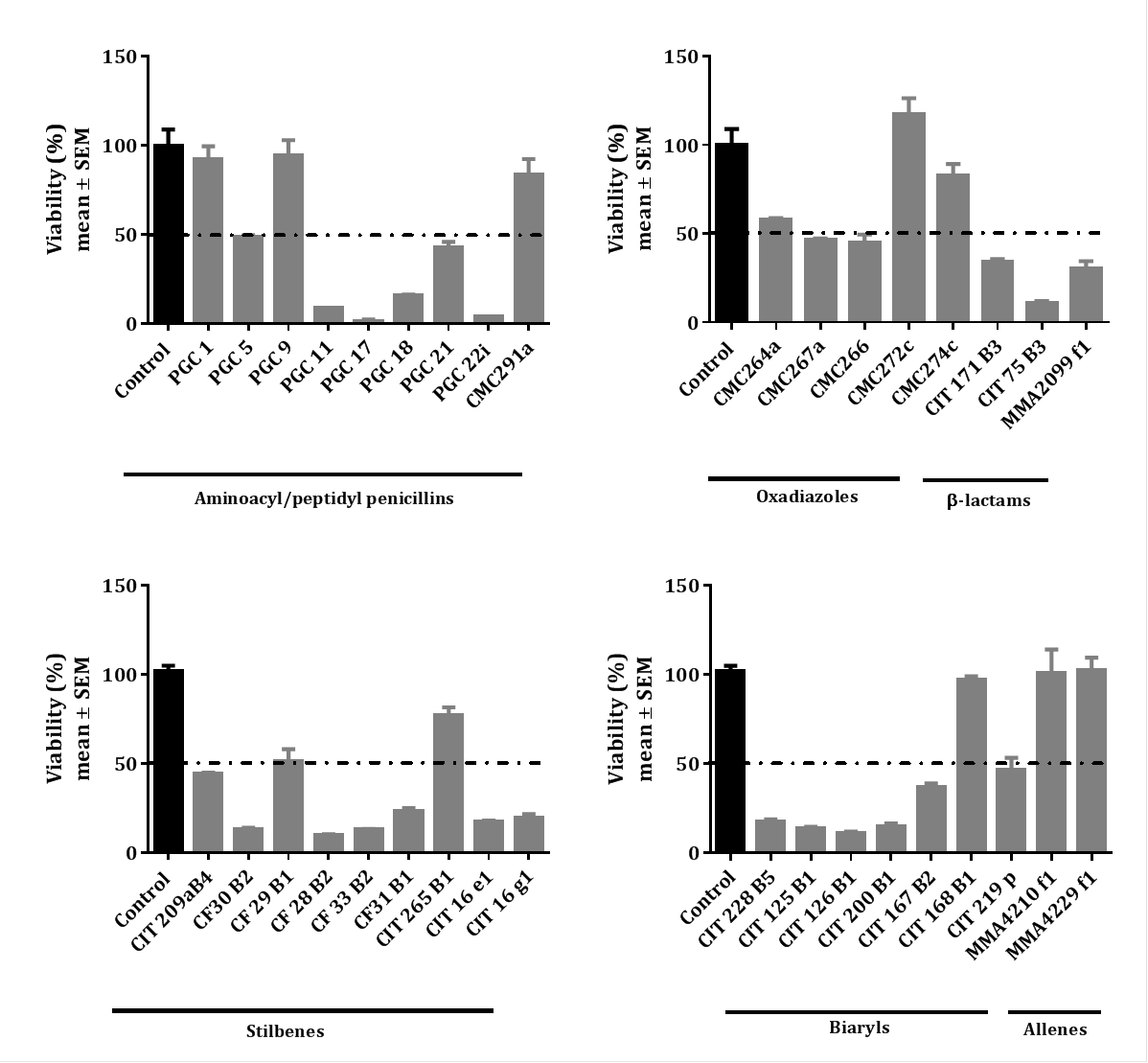

Supplement: Supplementary file 3 [file fsoa-06-442-s3.tif]

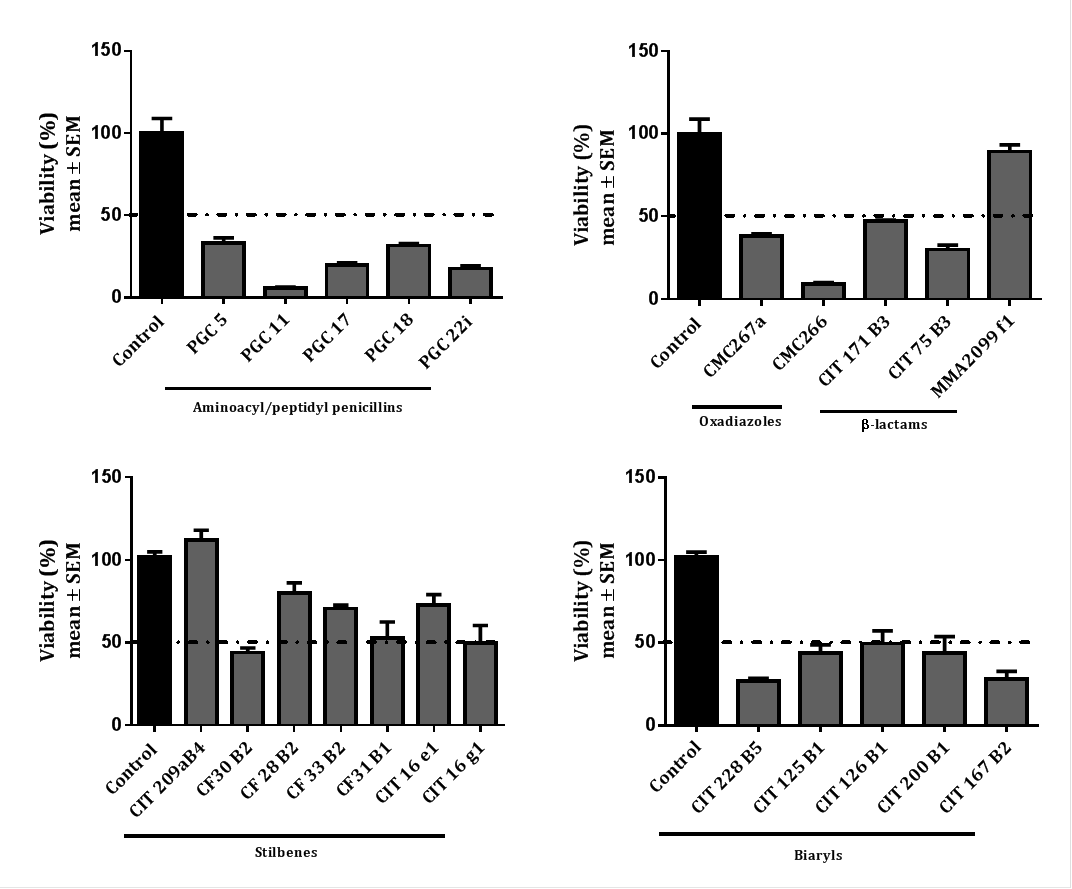

Supplement: Supplementary file 4 [file fsoa-06-442-s4.tif]

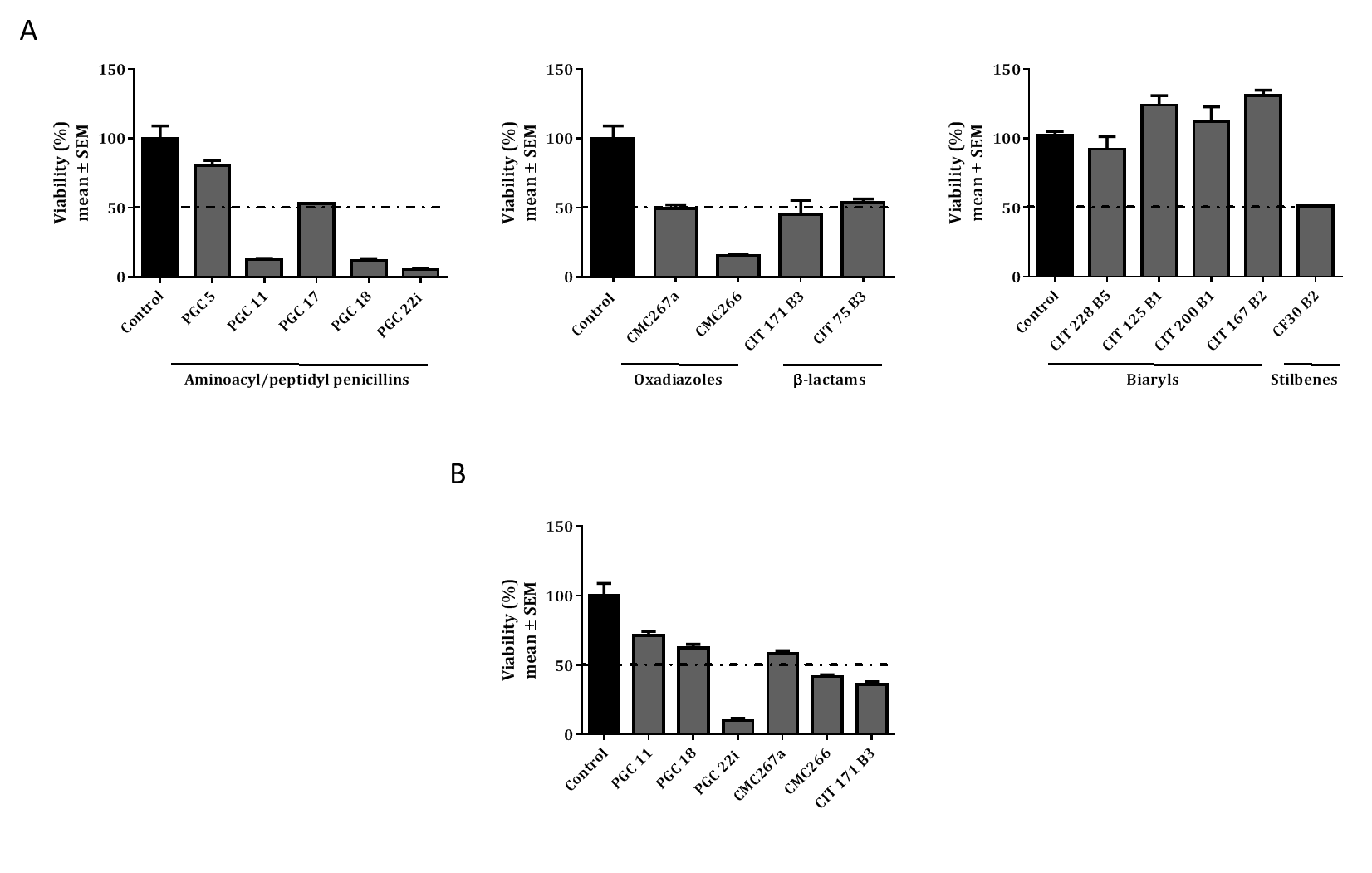

Supplement: Supplementary file 5 [file fsoa-06-442-s5.tif]
