# Supplementary material for: Antitumor activity of new chemical compounds in triple negative mammary adenocarcinoma models
Source: Future Sci OA. 2020 Jan 23;6(3):FSOA442. doi: 10.2144/fsoa-2019-0057 (PMC7050605; doi:10.2144/fsoa-2019-0057)
Supplement: Supplementary file 6 [file fsoa-06-442-s6.docx]

| **Compound** | **Percent of viability**  **mean ± SEM** |
| --- | --- |
| PGC11 | 71.62 ± 2.841 |
| PGC18 | 62.5 ± 2.614 |
| PGC22i | 10.51 ± 1.08 |
| CMC267a | 58.62 ± 1.875 |
| CMC266 | 41.68 ± 0.941 |
| CIT171B3 | 36.33 ± 1.752 |
